# BioRGroup dataset: R-group expansion of ChEBI molecules referenced in the Rhea database

**DOI:** 10.1038/s41597-025-05983-w

**Published:** 2025-10-27

**Authors:** Guillaume Gricourt, Jean-Loup Faulon

**Affiliations:** 1 Université Paris-Saclay, INRAE, AgroParisTech, Micalis Institute, 78350 Jouy-en-Josas, France; 2 The University of Manchester, Manchester Institute of Biotechnology, Manchester, M1 7DN UK

**Keywords:** Natural products, Chemical libraries, Computational chemistry

## Abstract

The application of artificial intelligence in cheminformatics highlights the necessity of comprehensive datasets that fully utilize all available chemical information. While generalist databases such as PubChem provide extensive compound coverage, specialised resources such as Rhea, which relies on the ChEBI ontology, are critical for the study of enzyme-catalysed reactions. A notable challenge arises from the presence of generic structures in ChEBI molecules, which incorporate R-groups as placeholders for various molecular fragments. This creates difficulties for their use in computational pipelines, such as those applied in retro-biosynthesis and biocatalysis. To address this issue, a curated dataset is presented that resolves R-group-containing ChEBI entries into fully defined molecular instances. The pipeline extracts generic molecules from Rhea, identifies compatible substitutions using PubChem and RDKit, and applies tailored filters to generate chemically valid enumerations. The dataset, BioRGroup, is delivered in a standard file format, thereby enabling the systematic integration of previously under-utilised generic structures into computational workflows, enhancing the scope and granularity of chemical data analysis.

## Background & Summary

The significance of data has become increasingly evident with the rise of artificial intelligence (AI). As AI continues to reshape scientific fields in cheminformatics, the availability of high-quality, structured data is essential for training models, generating insights, and guiding discovery. Typically, this data is organised in either general or specialised databases.

Generalist databases such as PubChem^1^ are essential resources for many applications in cheminformatics. Pubchem contains more than 100 million compounds from numerous contributors, covering a wide range of molecules, from small organic compounds to complex lipids, annotated with structural physicochemical, and bioactivity data, among others. However, in many research areas such as biocatalysis^2^ and retro-biosynthesis^3^, specialised databases like Brenda^4^, Reaxys^5^, and Rhea^6^ are essential. These databases focus on biochemical reactions, reactions catalyzed by enzymes, and often describe enzymatic transformations and transport processes. Many reactions involve natural products, defined as compounds produced by living organisms that are typically characterized by higher molecular weight, greater structural complexity, and a higher number of chiral centers than other organic molecules^7^. Chirality, in particular, has been identified as a key determinant of their bioactivity^8^. Relying on manual curation, these resources offer high quality data, albeit in limited quantities, with only a few tens of thousands of reactions.

It is notable that Rhea, a freely available resource, has a native implementation of the chemical ontology ChEBI^9^. Each Rhea reaction links the participating molecules to their corresponding ChEBI entries, providing a standardised framework for chemical classification and enabling semantic interoperability. Crucially, some of these ChEBI entries are generic chemical structures, defined using R-groups, placeholders that denote unspecified or variable molecular fragments. These generic forms allow broad representation of chemical families, but pose challenges for downstream chemoinformatics tasks, as they lack concrete molecular instances, an essential requirement for cheminformatic tools and AI models.

To support the validation of cheminformatics tools and the training of AI models, datasets are typically constructed and formatted for specific applications. Nevertheless, there is a paucity of research studies that explicitly address how R-groups are handled during the dataset preparation. For instance in biocatalysis, R-groups are substituted with simpler groups, such as methyl groups^10^, or are removed entirely^11^. Similarly, in the context of retro-biosynthesis workflows, reactions containing undefined atomic entities are usually excluded from datasets altogether^12^. While such exclusionary strategies may appear pragmatic at first glance, they inevitably result in the loss of chemically and biologically relevant information, thereby limiting the scope and accuracy of downstream analyses. To fully harness the potential of curated chemical resources in AI-driven research, it is therefore essential to bridge the gap between abstract chemical representations and usable molecular instances.

**Fig. 1:**
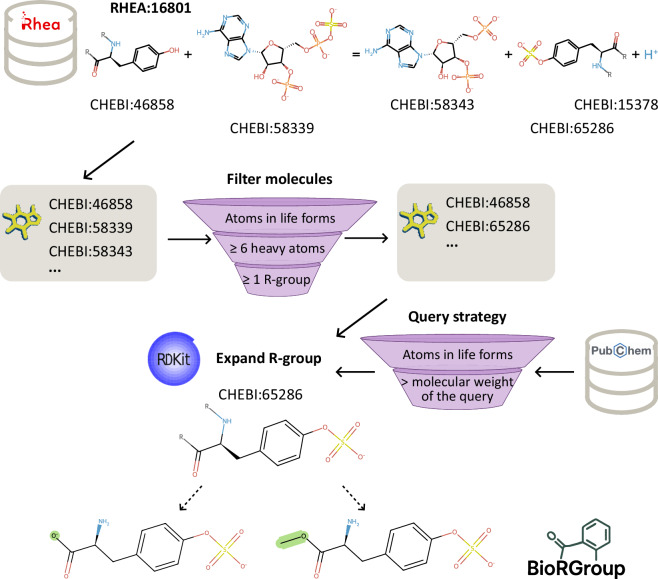
Overview of the R-group expansion process. The workflow starts with the Rhea reaction database, which provides substrates and products annotated with ChEBI identifiers. Molecules containing atoms commonly found in living organisms with at least one R-group and a minimum of six heavy atoms are selected. To create the BioRGroup dataset, R-group–containing templates were matched with PubChem molecules, expanding the R-group structures and linking them to fully defined molecular instances. In the molecules, R-groups are denoted by the character “R”, while atoms and bonds completing the undefined group are shown in green.

In this work, we present a curated dataset, BioRGroup, that systematically expands ChEBI entries containing R-groups into fully defined molecular instances. Our workflow (see Fig. 1) begins with the Rhea database, which serves as a source of biologically relevant query molecules. Each reaction in Rhea is annotated with ChEBI identifiers representing molecular participants, some of which correspond to generic molecules containing one or more R-groups. In many cases, these ChEBI entries are associated with SMILES strings, a line notation used to represent chemical structures^13^, including their generic components. To resolve these components into concrete chemical forms, we developed an enumeration pipeline using RDKit^14^ and molecular data from PubChem. For each R-group-containing ChEBI molecule, we identify all viable substitutions from PubChem that match the underlying chemical context, incorporating stereochemistry. By applying a set of tailored filters and optimisation criteria, we efficiently generate a comprehensive set of fully specified molecules while minimising computational cost.

The resulting dataset is provided as a gzip-compressed CSV file, for easy integration into cheminformatics workflows. This resource allows the systematic inclusion of generic ChEBI molecules in computational analyses, rather than excluding them due to their undefined nature.

## Methods

We downloaded the complete Compound database from PubChem via their FTP service (https://pubchem.ncbi.nlm.nih.gov, accessed on 2024/08/03). The data, originally provided in XML format, were integrated into an in-house SQL database, thereby ensuring the preservation of key molecular attributes including the Pubchem Compound Identifier (CID), molecular weight, SMILES, and InChI strings. The retention of molecules was contingent upon the successful parsing and validation of their InChI representations using RDKit, with the objective of ensuring structural consistency. Upon the receipt of queries, the database returned the corresponding InChI entries. To reduce computational overhead and focus on biologically relevant compounds, molecules containing elements that are rarely observed in natural systems were excluded from the dataset^15^. Accordingly, only molecules composed of hydrogen, carbon, nitrogen, oxygen, sodium, magnesium, phosphorus, sulfur, chlorine, potassium, or calcium were retained.

Next, we extracted the SMILES strings associated with CHEBI identifiers from the Rhea database (https://www.rhea-db.org, Release 134) using the file “rhea-chebi-smiles.tsv”. Using the same filtering strategy as applied to PubChem, we excluded molecules containing atoms that are not commonly found in living organisms. We further refined the selection by retaining only molecules with at least one R-group structure and a minimum of six heavy atoms in order to reduce computational requirements. An R-group structure refers to a placeholder atom attached to a core molecule, representing one or more possible substituents. For example, molecules with a generic R-group placeholder, such as CHEBI:12834 (*CCC = O) and CHEBI:22326 (*SO), were not retained because they contain fewer than six heavy atoms.

To identify real molecules sharing the same atomic core, we selected candidate molecules from the PubChem database whose molecular weights exceeded those of the corresponding ChEBI compounds. For this task, we used the rdRGroupDecomposition^16^ module of RDKit (version 2024.9.3), which enables the identification of non-terminal R-groups and, by default, accounts for chiral centers and double-bond configurations, but not for enhanced stereochemical representations. Furthermore, the search was not extended to encompass the entirety of the possible tautomers of molecules that contain R-groups. A timeout of 10 seconds per molecule was imposed to manage computational resources efficiently. This constraint ensured practical run-time limits while maintaining a high coverage rate. As a single PubChem InChi entry may represent multiple molecular entities, a secondary R-group decomposition step was applied to disaggregate these composite entries and isolate relevant matches. However, the default behaviour of the rdRGroupDecomposition module includes consideration of additional substituents beyond the specified R-group position. To mitigate this, we introduced a third step to retain only those molecules in which a single atom or group was added exclusively at the R-group location specified in the original query molecule. To maintain traceability, the CID of the original PubChem entry associated with the original match was retained and reported alongside the decomposed results.

The first phase of the R-group search was a major computational bottleneck. On average, the decomposition process took approximately two hours to complete when running on twelve CPU cores on a high performance computing cluster. Efficient distribution and scheduling of this workload across cluster nodes were achieved using Snakemake^17^, which provided a scalable workflow management system to orchestrate the parallel execution of the R-group expansion pipeline.

## Data Record

**Fig. 2:**
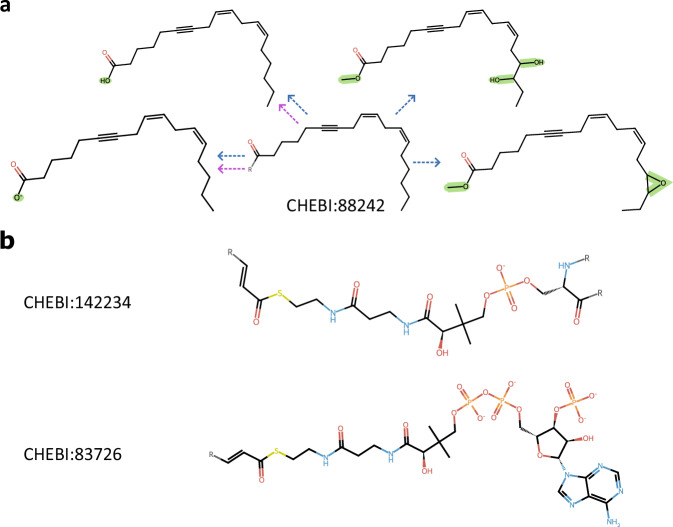
Dataset examples. (**a**) Example of core structure matching: four molecules sharing the core structure of ChEBI entry 88242 are connected by blue arrows and are listed in the column “core_superstructure_smiles”. Two molecules sharing the same core structure but differing only at the R-group position are connected by purple arrows and are listed in the column “rgroup_extended_smiles”. Additional bonds and atoms are shown in green. (**b**) Examples of molecules for which no matching molecules were found in PubChem.

The dataset, BioRGroup, is available at Recherche Data Gouv (https://recherche.data.gouv.fr^, version 2.2)18^. BioRGroup, described in Table 1, is provided as a gzip-compressed CSV file containing eight columns and 12,709 records. The “smiles” and “chebi” columns contain the same molecular data as found in the Rhea database, from the file “rhea-chebi-smiles.tsv”, with the important distinction that the “chebi” field may contain multiple ChEBI identifiers, representing cases where the same SMILES string corresponds to multiple entries. Two additional columns, “num_heavy_atoms” and “exact_mol_wt”, report key molecular properties used as criteria in the analysis: the number of heavy atoms and the exact molecular weight of each molecule, both computed using RDKit. In addition, four supplementary columns provide essential information for the core structure matching analysis. The “core_superstructure_smiles” and “rgroup_extended_smiles” columns list molecules that match the core structure of the reference molecule, either with or without additional substituents, respectively, as shown in Fig. 2a. Corresponding PubChem compound identifiers for these matched molecules are provided in the columns “core_superstructure_pubchem_cid” and “rgroup_extended_pubchem_cid”. In some cases, no molecules were found that expanded the core structure of the ChEBI molecule (Fig. 2b). These cases are represented as an empty list in the CSV file.

**Table 1:** Content of the BioRGroup dataset.

Column name	Type	Description
smiles	String	SMILES of the ChEBI molecule
chebi	List[String]	List of ChEBI identifiers sharing the same SMILES
num_heavy_atoms	Integer	Number of heavy atoms retrieved in the molecule
exact_mol_wt	Float	Molecular weight of the molecule
core_superstructure_smiles	List[String]	SMILES of the molecule retrieved from PubChem that match the core structure of the molecule, allowing for the possibility that atoms are not only present at the R-group sites.
core_superstructure_pubchem_cid	List[List[Integer]]	PubChem identifiers of the molecules. A single molecule can have one or more PubChem identifiers.
rgroup_extended_smiles	List[String]	SMILES of the molecule retrieved from PubChem that match the core structure of the molecule, only at the R-group locations.
rgroup_extended_pubchem_cid	List[List[Integer]]	PubChem identifiers of the molecules. A single molecule can have one or more PubChem identifiers.

The dataset is provided as a gzip-compressed CSV file and extends the Rhea database by integrating additional annotations, including descriptive features, the SMILES of molecules matching the core structure, and their PubChem identifiers.

## Technical Validation

Qualitative and quantitative descriptions, as well as technical validations, were performed on the two source datasets: PubChem and Rhea, and on the resulting R-group expansion dataset to assess whether they were properly filtered and contained the expected data.

### PubChem dataset

The dataset downloaded from PubChem was used to create an SQL database containing 118,565,503 entries. Of these, 83,437,906 records contain only atoms that are commonly found in living organisms, according to Alberts *et al*.^15^. Magnesium is the least common atom, while hydrogen is the most common, with a difference of five orders of magnitude. Given the scale of the dataset, we believe it is sufficient to capture the chemical diversity of the molecules under investigation.

### Rhea dataset

The Rhea database contains 12,962 records that associate a ChEBI identifier, used in reactions in the database, with its corresponding SMILES representation. After merging ChEBI identifiers that share identical SMILES, the dataset was reduced to 12,709 unique entries. Of these, 10,585 molecules contain no R-groups, while 2,124 molecules contain at least one R-group. In addition, 119 molecules containing fewer than six heavy atoms were excluded. Finally, 15 molecules containing atoms not commonly found in living organisms were discarded.

After applying these filters, more than 93.7% of the molecules with at least one R-group were retained. This is considered to be sufficient to adequately represent the original dataset.

### Expansion dataset

First, we evaluated the impact of the 10-second timeout applied during the identification of non-terminal R-groups. To assess its effect on the detection of molecules that share a core structure and to ensure coverage across the full molecular weight range, 50 molecules were selected at regular intervals between the minimum and maximum weight. Timeouts of 2, 10 and 20 seconds were used to identify molecules sharing a core structure. In all cases, coverage exceeded 99.99% (99.99988%, 99.99994% and 99.99995% respectively). With a 20-second timeout, only 18 molecules exhibited any timeout against PubChem entries. For these cases, an average of four entries were not evaluated under the 2-second condition and one entry under the 10-second condition. However, none of the entries that timed out at 2 or 10 seconds matched the corresponding core structure. These results suggest that a 10-second timeout is sufficient to achieve near-complete coverage while maintaining computational efficiency.

Next, we compared the properties of the molecules in the BioRGroup dataset with those in several reference collections. These were the eMolecules database (33,648,359 commercially available compounds, https://www.emolecules.com, accessed August 2025), ChEMBL^19^ (2,474,576 bioactive drug-like molecules, https://www.ebi.ac.uk/chembl, Release 35), MetaNetX^20^ (1,243,158 natural compounds from genome-scale metabolic networks and biochemical pathways, https://www.metanetx.org, Release 4.4) and 17,182,035 molecules retrieved from PubChem and expanded only at R-group positions.

**Fig. 3:**
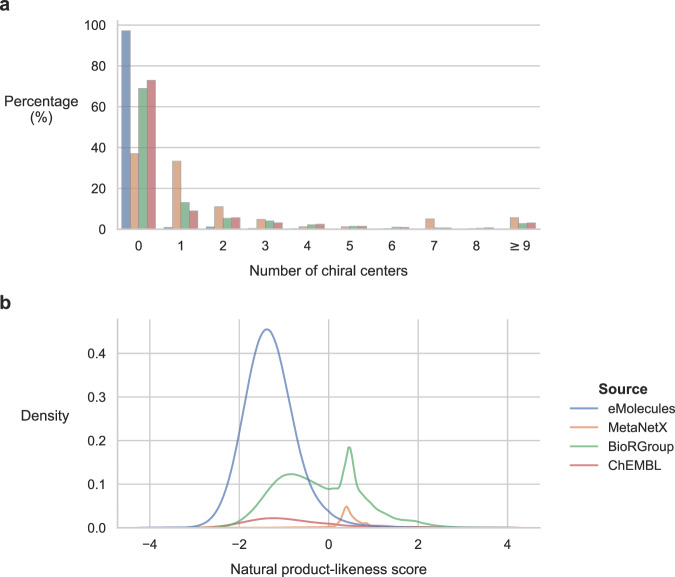
Property distributions of molecules from MetaNetX, ChEMBL, eMolecules, and BioRGroup (expanded only at the R-group locations). (**a**) Frequency distribution of defined chiral centers. (**b**) Probability density distributions of natural product-likeness scores.

We examined the distribution of defined chiral centers (Fig. 3a), computed using RDKit. The majority of eMolecules compounds lacked chiral centers (97.2%), whereas BioRGroup and ChEMBL displayed similar patterns, with 31.1% and 27.1% of molecules containing at least one chiral center, respectively. MetaNetX showed an even higher proportion, with 63.0% of molecules containing at least one chiral center. Table 2 also shows that the average number of chiral centers per molecule in BioRGroup (2.0) is close to that in ChEMBL (1.4), but higher than in synthetic molecules (0.4) and lower than in natural products (5.2). A comparable trend in the ratio between natural products, drugs, and synthetic molecules was previously observed by Feher and Schmidt^21^.

**Table 2:** Average number of chiral centers for each molecule from MetaNetX, ChEMBL, eMolecules, and BioRGroup (expanded only at the R-group locations).

	MetaNetX	ChEMBL	eMolecules	BioRGroup
Defined chiral centers	2.8	1.1	0.1	1.1
Defined/Undefined chiral centers	5.2	1.4	0.4	2.0

We then assessed the natural product-likeness of the dataset using the established NP-likeness score^22^, which evaluates the similarity of a molecule to the structural space of natural products based on its molecular fragments, with higher values indicating stronger resemblance. As illustrated in Fig. 3b, the BioRGroup dataset exhibits a bimodal distribution, with one peak overlapping with eMolecules and ChEMBL and the other aligning with MetaNetX. These findings indicate that BioRGroup encompasses both natural product-like and non-natural product-like chemical spaces, and that NP-likeness score can be used to identify molecules of interest, such as those involved in biochemical reactions.

## Usage Notes

We have deliberately provided the data in CSV format to ensure maximum interoperability across platforms and tools. However, due to the size of the file and the complexity of the data types it contains, parsing the dataset can be challenging. To assist users, a Jupyter Notebook is available in the GitHub repository that demonstrates how to parse and interact with the data using Python.

Users can regenerate or update the dataset using the provided code to incorporate more recent sources. Currently, there is no mechanism in place to restrict updates to newly added molecules only. Therefore, the entire process must be run again to obtain an updated dataset.

## Data Availability

The BioRGroup dataset, which is provided as a gzipped CSV file together with its associated metadata, is available at Recherche Data Gouv (10.57745/V3URYA)^18^.

## Code availability

The code used to download and build the Pubchem and Rhea databases, the code used to expand the R-group, and the code used to construct the figures are available in this GitHub repository: https://github.com/brsynth/biorgroup.
